# Relationship Between Motor Capacity of the Contralesional and Ipsilesional Hand Depends on the Side of Stroke in Chronic Stroke Survivors With Mild-to-Moderate Impairment

**DOI:** 10.3389/fneur.2019.01340

**Published:** 2020-01-08

**Authors:** Rini Varghese, Carolee J. Winstein

**Affiliations:** ^1^Division of Biokinesiology and Physical Therapy, University of Southern California, Los Angeles, CA, United States; ^2^Department of Neurology, Keck School of Medicine, University of Southern California, Los Angeles, CA, United States

**Keywords:** ipsilesional deficits, stroke, hemispheric differences, upper limb, motor capacity

## Abstract

There is growing evidence that after a stroke, sensorimotor deficits in the ipsilesional hand are related to the degree of impairment in the contralesional upper extremity. Here, we asked if the relationship between the motor capacities of the two hands differs based on the side of stroke. Forty-two pre-morbidly right-handed chronic stroke survivors (left hemisphere damage, LHD = 21) with mild-to-moderate paresis performed distal items of the Wolf Motor Function Test (dWMFT). We found that compared to RHD, the relationship between contralesional arm impairment (Upper Extremity Fugl-Meyer, UEFM) and ipsilesional hand motor capacity was stronger ($R_{LHD}^{2}=$ 0.42; $R_{RHD}^{2}$ < 0.01; *z* = 2.12; *p* = 0.03) and the slope was steeper (*t* = −2.03; *p* = 0.04) in LHD. Similarly, the relationship between contralesional dWMFT and ipsilesional hand motor capacity was stronger ($R_{LHD}^{2}=$ 0.65; $R_{RHD}^{2}$ = 0.09; *z* = 2.45; *p* = 0.01) and the slope was steeper (*t* = 2.03; *p* = 0.04) in LHD compared to RHD. Multiple regression analysis confirmed the presence of an interaction between contralesional UEFM and side of stroke (β_3_ = 0.66 ± 0.30; *p* = 0.024) and between contralesional dWMFT and side of stroke (β_3_ = −0.51 ± 0.34; *p* = 0.05). Our findings suggest that the relationship between contra- and ipsi-lesional motor capacity depends on the side of stroke in chronic stroke survivors with mild-to-moderate impairment. When contralesional impairment is more severe, the ipsilesional hand is proportionally slower in those with LHD compared to those with RHD.

## Introduction

It is now well-known that unilateral stroke not only results in weakness of the opposite half of the body, i.e., contralateral to the lesion or contralesional limb, but also significant motor deficits in the same half of the body, i.e., ipsilateral to the lesion or ipsilesional limb (1–4). Previous work suggests that deficits in the ipsilesional arm and hand varies with the severity of contralesional deficits, especially in the sub-acute and chronic phase after stroke (5–8). More interestingly, the unilateral motor deficits observed for contralesional and ipsilesional limbs seem to be hemisphere-specific and thus depend on *side* of stroke lesion (9–15). For predominantly right-handed cohorts, contralesional deficits appear to be more severe in those with right hemisphere damage (RHD), in whom the contralesional limb is non-dominant. For example, using clinical motor assessments of grip strength and hand dexterity, Harris and Eng (11) showed that contralesional motor impairments were less severe in chronic stroke survivors who suffered damage in the dominant (i.e., left) hemisphere (LHD) compared to those who suffered damage in the non-dominant (right) hemisphere (11, 15).

In contrast, considering ipsilesional motor deficits, the evidence is mixed concerning hemisphere-specific effects. For instance, some studies reported that individuals with LHD exhibited more severe ipsilesional arm and hand deficits compared to those with RHD (4, 15–17) while others have reported no difference in ipsilesional hand motor capacity between LHD and RHD (2). In acute stroke survivors, Nowak et al. demonstrated that deficits in grip force of the ipsilesional hand were significantly associated with clinical measures of function of the contralesional hand *only* in LHD (12). Contrary to this, de Paiva Silva et al. (14) found that compared to controls and LHD, the ipsilesional hand in chronic stroke survivors was significantly slower and less smooth in RHD especially when contralesional impairment was relatively more severe (UEFM < 34).

**Figure 1:**
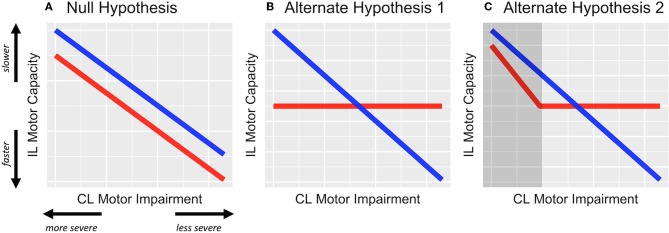
Hypothesized effects represented in schematic figure. **(A)** The null hypothesis, wherein the relationship between contralesional (CL) impairment and ipsilesional (IL) motor capacity is not modified by the side of stroke lesion, i.e., β_1_ ≠ 0 but β_3_ = 0. **(B)** Alternative hypothesis 1, wherein ipsilesional deficits are related to contralesional impairment but only in LHD (blue) and not in RHD (red). **(C)** Alternate hypothesis 2, wherein ipsilesional deficits are related to contralesional impairment but only in LHD and in RHD with severe impairment (represented in the shaded dark-gray area). For both alternate hypotheses, β_1_ and β_3_ ≠ 0.

Taken together, there is converging evidence regarding the relationship between motor deficits of the contralesional and ipsilesional upper extremity, such that ipsilesional deficits are worse when contralesional impairment is greater (Figure 1A); however, it is uncertain whether the relationship between the two limbs depends on which hemisphere is damaged. In particular, motor deficits of the two limbs are most prominent for tasks that require dexterous motor control (e.g., grip force, tapping, tracking). For predominantly right-handed cohorts (as is the case in most studies), contralesional deficits appear to be more severe in those with RHD, in whom the contralesional limb is non-dominant; whereas ipsilesional deficits are more severe in those with LHD. An exception to this observation for those with RHD seems to be in the case when contralesional impairment is most severe (i.e., UEFM < 34) (14). Thus, one might predict that as contralesional impairment worsens, individuals with LHD would have proportionally worse ipsilesional deficits, but individuals with RHD (especially if say UEFM > 34) would not; see Figures 1B,C for two alternative hypotheses. To our knowledge, this prediction has not before been explicitly tested.

One reason that this prediction remains untested might be methodological in that in these previous studies participants were categorically classified based on the degree of contralesional motor impairment (e.g., mild, moderate, severe) (6, 8, 14). Categorization (or worse, dichotomization) of a continuous variable presents several concerns, including loss of measurement resolution, an assumption of discontinuity in the underlying construct (in this case motor impairment), unequal subgroup sizes (or biased sampling), and large unexplained residuals in regression models (18–20). Overall, if the objective is to understand the nature and extent of critical response-predictor relationships, then a categorical approach is particularly problematic.

Our primary objective was to determine if the severity of deficits in the ipsilesional hand varies directly with that of the contralesional hand and if this relationship differs based on the side of stroke lesion (i.e., an interaction effect). To accomplish this objective, we conducted a retrospective regression analysis of an existing dataset taken from mild-to-moderately impaired chronic stroke survivors. Although the range of contralesional impairment was limited in this dataset, we preserved its continuity and tested the prediction that the dexterous motor capacity of the ipsilesional hand varies directly with the severity of the contralesional motor impairment in individuals with LHD, but not in individuals with RHD (see Figure 1).

## Methods

### Participants

A retrospective analysis of data from 42 chronic stroke survivors (*n* = 21 left-hemisphere damage, LHD) was conducted. Participants were enrolled as part of a larger phase-IIb clinical trial (Dose Optimization for Stroke Evaluation, ClinicalTrials.gov ID: NCT01749358) (21) and provided informed consent in accordance with the 1964 Declaration of Helsinki and the guidelines of the Institutional Review Board for the Health Science Campus of the University of Southern California. Participants were at least 150 days post-stroke, pre-morbidly right-handed with mostly resolved upper extremity paresis. For a complete description of the inclusionary and exclusionary criteria, please refer to Supplementary Materials.

### Outcome Measures

#### Motor Component of the Upper Extremity Fugl-Meyer (UEFM)

The UEFM (22) is an assessment of motor impairment of the contralesional arm and hand after stroke and includes tests of strength and independent joint control. Item-wise scoring of the UEFM ranges from 0 (unable to perform) to 2 (able to perform completely) while total score ranges from 0 to 66, with a higher score indicating lesser impairment. The UEFM score was modeled as a continuous variable for statistical analysis purposes.

#### Wolf Motor Function Test (WMFT)

The WMFT assesses upper extremity motor capacity through timed functional task performance (e.g., lifting a can, pencil, or paper clip). Originally designed for patients with moderate to severe upper extremity motor deficits, the test was later modified by Morris, Crago, and Taub to accommodate individuals with mild motor impairments (23). Hand motor capacity was assessed for both the contralesional and ipsilesional hands using the distal task battery of the WMFT (dWMFT) (24) which consisted of the following 8 tasks: lift can, lift pencil, lift paper clip, stack checkers, flip cards, turn a key in a lock, fold towel, and lift basket. A Principal Component Analysis of WMFT time scores revealed two clusters: one consisting of the proximal (#1–8, except 6, i.e., lifting weight to box), the other consisting of the distal (#9–17, except 14, i.e., grip strength) (Kim et al., unpublished, personal communication).

### Statistical Analyses

All analyses were conducted in the R statistical computing package version 3.5.1 (25). To test the hypothesis that the inter-limb relationship of motor capacity is modified by the side of stroke lesion, we used the coefficient of determination (*R*^2^) and compared the covariances between LHD and RHD using the Fisher's *Z*-test. We then performed a simple linear regression to determine the slope of the relationship between contralesional (CL) UEFM and ipsilesional (IL) dWMFT (Model 1), and, CL dWMFT and IL dWMFT (Model 2). We used *t*-tests to compare slope between LHD and RHD.

To supplement these primary analyses and as a more robust assessment of the interaction between the side of lesion and contralesional motor capacity, we used multiple linear regression of the following form:

$$\begin{array}{l} \text{Mode}l\;1:\;y=\;\beta_{0}+\;\beta_{1}\left(CL\;UEFM\right)+\;\beta_{2}\left(Side\;of\;Stroke\right)\\ \;+\beta_{3}\left(CL\;UEFM*Side\;of\;Stroke\right)+\epsilon \\ \text{Mode}l\;2:\;y=\;\beta_{0}+\;\beta_{1}(CL\;dWMFT)+\;\beta_{2}(Side\;of\;Stroke)\\ \;+\;\beta_{3}(CL\;dWMFT*Side\;of\;Stroke)+\epsilon \end{array}$$

In both models, *y* is the average time score on the distal WMFT of the ipsilesional hand. Using this multiple model, our hypotheses were that β_1_ ≠ 0 and β_3_ ≠ 0 (see Figure 1). Any statistically significant interaction was resolved *post-hoc* using a *t*-test comparison of estimated marginal trends between LHD and RHD.

All continuous variables were assessed for normality using Lilliefors test (modified Kolmogorov–Smirnov test). Distributions for chronicity and average time-score for the distal WMFT were positively skewed and were therefore log-transformed. Welch's *t*-tests were used to compare age, chronicity, and Upper Extremity Fugl-Meyer scores between LHD and RHD, whereas Chi-square test was used to compare the proportion of females and males between the two groups. Each group was standardized to its own unit variance (z-scored) to equalize range and for subsequent linear regression analysis. Outliers were identified by visual inspection of scatterplots. Any value of IL dWMFT more extreme than ±1.5 log-SD was examined carefully for their influence on interlimb covariance and slope. If removal of these observations did not change the direction or significance of the effect in the simple model, we included them in the final model. Residuals of the final model were analyzed to confirm that all necessary assumptions for multiple regression were met. Significance level (α) was set at *p* < 0.05.

In order to select the predictor variables that best explain the response, we used a backward selection approach, in which we began by adding all predictor variables in each of the two above models to explain the response variable *y*. This included our hypothesized predictors, CL UEFM (or CL dWMFT) and the side of stroke lesion (LHD or RHD), and, potential known confounders (age, chronicity, and sex). In a combined full model, those predictors that met a liberal cut-off of *p* = 0.2 were preserved in the final reduced model. Based on this selection process, we found sex to be a significant confounder (*p* = 0.08) in Model 1, and therefore included it as a predictor in the reduced Model 1. For Model 2, none of the confounders met the cut-off *p*-value, except our hypothesized predictors. Additional information on model selection and model diagnostics is included as Supplementary Materials. Standard errors and 95% CI of the estimates of regression coefficients were confirmed by performing 1,000 bootstrap replicates.

## Results

Descriptive statistics for all participants are provided in Table 1. On average, the 42 stroke survivors had moderate arm impairment (UEFM = 41.6), were ~60 years of age, 5.75 years post-stroke, and were predominantly male (74%). There were no significant differences between LHD and RHD in the level of impairment, chronicity, or the number of males. Individuals with RHD were younger compared to LHD (median age difference 8.7 years) but not statistically different.

**Table 1:** Descriptive Statistics for the full sample (*N* = 42), and for the two groups of interest, left hemisphere damage, LHD (*n* = 21), and right hemisphere damage, RHD (*n* = 21).

**Variable**	**Overall (*N* = 42)** Mean ^*^ (± SD)	**LHD (*n* = 21)** **Mean (± SD)**	**RHD (*n* = 21)** **Mean (± SD)**	**two-sided** ***p*-value**
Sex (Male)^*^	31 (73.8)	15 (71.4)	16 (76.2)	~1
Age (years) [Min, Max]	59.16 (12.3) [35.48, 80.54]	60.71 (10.8) [43.15, 80.54]	57.62 (13.8) [35.48, 77.28]	0.42
Chronicity (months) [Min, Max]	69.37 (36.9) [26.33, 212.52]	63.32 (24.4) [30.02, 111.52]	75.41 (46.1) [26.33, 212.52]	0.29
UEFM Motor (/66) [Min, Max]	42 (10) [19, 59]	41 (13) [19, 59]	42 (8) [28, 55]	0.58

* *Count (percent) for categorical variables*.

### Model 1: Side of lesion Modifies the Relationship Between CL UEFM and IL Motor Capacity

Contralesional UEFM explained 42% of the variance in ipsilesional hand motor capacity in LHD (*p* = 0.001), but <1% in RHD (*p* > 0.05). The slope of this relationship was −0.65 ± 0.17 (*p* = 0.001) in LHD and −0.066 ± 0.23 (*p* = 0.78) in RHD. Compared to RHD, the covariance between contralesional UEFM and ipsilesional hand motor capacity was significantly stronger (Fisher's *z* = 2.12, *p* = 0.03) and the slope was steeper in LHD (*t* = −2.03, *p* = 0.04).

Four observations (two each in LHD and RHD) were identified as potential outliers. After removal of these outliers, contralesional UEFM explained 44.3% of the variance in ipsilesional hand motor capacity in LHD (*p* = 0.001), and 2.26% in RHD (*p* > 0.05). The slope of this relationship changed to −0.42 ± 0.11 (*p* = 0.001) in LHD and 0.13 ± 0.20 (*p* = 0.54) in RHD. Again, a comparison of the covariances and slope between the groups revealed that compared to RHD, the relationship between contralesional UEFM and ipsilesional hand motor capacity was significantly stronger (Fisher's *z* = 2.7, *p* = 0.006) and the slope was steeper in LHD (*t* = −2.41, *p* = 0.02).

Since these observations did not significantly change the strength of covariance nor the slope of the relationship, they were preserved in the final multiple model. Analysis of residuals of the final model did not indicate violations of necessary assumptions in multiple regression in terms of linearity, equality of variance, independence and normality of errors, and multicollinearity of independent variables, nor the presence of unduly influential observations. Nonetheless, estimates below are reported both with and without suspected outliers.

**Figure 2:**
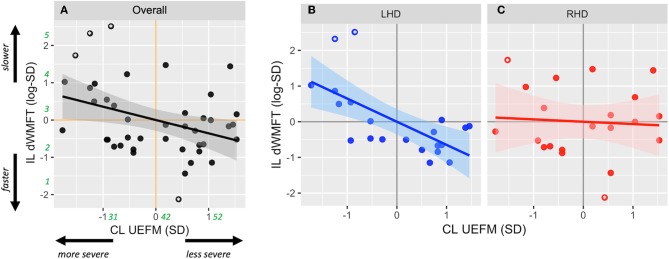
Scatterplots show the relationship between contralesional motor impairment (CL UEFM) and ipsilesional distal motor performance (IL dWMFT) for **(A)** the full sample, **(B)** LHD, and **(C)** RHD. Solid lines represent the linear prediction and shaded areas represent the 95% confidence interval. For ease of interpretation, rounded estimates of raw values (in seconds for dWMFT and points for UEFM) have been provided in green in **(A)**. Asterisks indicate values evaluated as outliers.

After adjusting for main effects and significant confounders using multiple regression, the final reduced form of Model 1 was statistically different from a null model (*F* = 3.47, *p* = 0.016, adjusted *R*^2^ = 0.19). Based on estimates from Model 1, CL impairment (UEFM) was significantly associated with IL hand motor capacity, i.e., dWMFT (β_1_ = −0.72 ± 0.21, *p* = 0.001; *without outliers*: −0.44 ± 0.17, *p* = 0.01) (Figure 2A). There was no significant effect of the side of lesion (β_2_ = 0.026 ± 0.27, *p* = 0.92; *without outliers*: 0.22 ± 0.23, *p* = 0.33). There was a significant interaction between the side of lesion and CL impairment (β_3_ = 0.66 ± 0.30, *p* = 0.024; *without outliers*: 0.56 ± 0.24, *p* = 0.024). *Post-hoc* contrasts of estimated marginal trends indicated that the slope of the relationship between CL UEFM and IL dWMFT was significantly more negative in LHD compared to RHD (*t* = −2.34, *p* = 0.02; *without outliers*: −2.37, *p* = 0.02). Figures 2B,C illustrates the interaction.

### Model 2: Side of lesion Modifies the Relationship Between CL dWMFT and IL Motor Capacity

Contralesional dWMFT explained 65% of the variance in ipsilesional hand motor capacity in LHD (*p* < 0.001), but only 9% in RHD (*p* > 0.05). The slope of this relationship was 0.81 ± 0.13 (*p* < 0.001) in LHD and 0.29 ± 0.22 (*p* = 0.19) in RHD. A comparison of the covariances and slope between LHD and RHD revealed that compared to RHD, the relationship between CL dWMFT and IL motor capacity was significantly stronger (Fisher's *z* = 2.45, *p* = 0.01) and the slope was steeper in LHD (*t* = 2.03, *p* = 0.04).

After removing the outlying observations, contralesional dWMFT explained 62% of the variance in ipsilesional hand motor capacity in LHD (*p* < 0.001), and <1% in RHD (*p* > 0.05). The slope of this relationship changed to 0.54 ± 0.1 (*p* < 0.001) in LHD and 0.05 ± 0.21 (*p* = 0.81) in RHD. Compared to RHD, the relationship between CL dWMFT and IL motor capacity was significantly stronger (Fisher's *z* = 2.85, *p* = 0.004) and the slope was steeper in LHD (*t* = 2.11, *p* = 0.04).

Since these observations did not significantly change the strength of covariance or the slope of the relationship, they were preserved in the final multiple model. Once again, analysis of residuals did not indicate violations of necessary assumptions in multiple regression nor the presence of unduly influential observations. Nonetheless, estimates below are reported both with and without suspected outliers.

**Figure 3:**
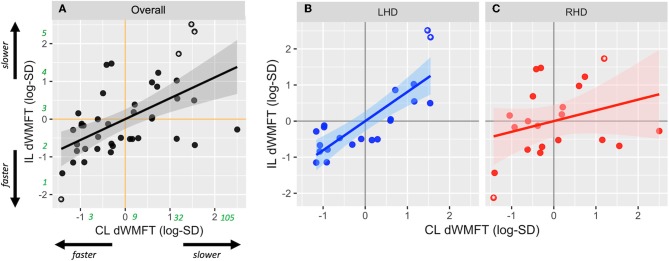
Scatterplots show relationship between contralesional distal motor performance (CL dWMFT) and ipsilesional distal motor performance (IL dWMFT) for **(A)** the full sample, **(B)** LHD, and **(C)** RHD. Solid lines represent the linear prediction and shaded areas represent the 95% confidence interval. For ease of interpretation, rounded estimates of raw values (in seconds) have been provided in green in **(A)**. Asterisks indicate values evaluated as outliers.

After adjusting for main effects and significant confounders using multiple regression, the final reduced form of Model 2 was statistically different from a null model (*F* = 7.48, *p* < 0.001, adjusted *R*^2^ = 0.32). Based on estimates from Model 2, CL hand motor capacity was significantly associated with IL hand motor capacity (β_1_ = −0.81± 0.16, *p* < 0.001; *without outliers*: 0.54 ± 0.17, *p* = 0.003) (Figure 3A). There was no significant effect of the side of lesion (β_2_ = 0.12 ± 0.26, *p* = 0.66; *without outliers*: 0.19 ± 0.22, *p* = 0.39). There was an interaction between the side of lesion and CL hand motor capacity, but it only approached statistical significance (β_3_ = −0.51 ± 0.34, *p* = 0.05; *without outliers*: −0.49 ± 0.24, *p* = 0.046). Figures 3B,C illustrates the interaction.

Finally, we note here that we conducted similar analyses for the proximal component of the WMFT (not reported) but did not observe the same relationships (model adj. *R*^2^ = ~3% for both models, *p* > 0.25). One possibility is that unlike the distal component, the proximal WMFT is a much less sensitive metric of motor performance, especially in individuals with mild-to-moderate impairment.

## Discussion

For the first time, we explicitly tested the hypothesis that motor capacity of the ipsilesional hand is influenced by an interaction between the severity of contralesional deficits and the side of stroke lesion. Using retrospective analysis of an existing dataset, we found that ipsilesional motor capacity co-varies with contralesional impairment to a significantly greater degree in individuals with LHD compared to RHD.

### Analysis of the Interaction Effect

Hints of this interaction were implicit in a few previous studies (2, 3, 14); however, categorical reporting of the Upper Extremity Fugl-Meyer (UEFM) masked this interesting effect. By preserving the continuity of the UEFM and utilizing continuous standardized z-scores, our statistical approach allowed a direct comparison of our regression estimates, thus reflecting effect sizes of each of the candidate predictors. Unlike previous studies, we did not observe a significant effect of the side of lesion on ipsilesional motor capacity (16, 17) or on contralesional UEFM or dWMFT (11). One reason might be that the effect of the side of lesion observed in those previous studies may have arisen from its interaction with contralesional impairment. However, because an interaction effect was not explicitly tested and because contralesional impairment was either collapsed across the groups (2, 3) or categorical (8, 14), variance in the ipsilesional capacity may have been conflated with the effect of the side of lesion, or, remained unexplained, especially for UEFM scores that fell at the boundaries of the pre-defined categories. Previously, various cut-off scores for the UEFM have been used to define impairment categories (8, 14). Of these, our data suggest that a UEFM score of 42, which occurs at the intersection of the linear fits for LHD and RHD, would best reflect the change in the direction of effect—that is, for UEFM scores <42, the ipsilesional limb would be slower in LHD compared to RHD whereas for UEFM scores >42, those with LHD would be slightly faster compared to RHD. Interestingly, *at* this score of 42, there would appear to be no differences in motor capacity of either hand between LHD and RHD, which might explain why a number of large clinical trials [e.g., EXCITE (26), ICARE (27)], designed for mild to moderately impaired stroke survivors (mean UEFM scores in these studies were 42.5 and 41.6, respectively) may not have observed, on average, any differences in motor capacity based on the side of stroke.

On a related note, in Model 1, we observed that the relationship between contralesional UEFM and ipsilesional motor performance was (mildly) confounded by sex. Specifically, males showed slightly faster performance times (mean difference = 0.3 s, β = −0.56, *p* = 0.08) compared to females. Given that this difference was very small and may have been exaggerated by the unequal sample sizes, i.e., there were about 3 times more males than females, in LHD, RHD and overall, we suspect this was an artifact of the unequal sample sizes rather than a true difference between males and females.

### Insights From the Type of Task

A common link between our study and past reports is that ipsilesional deficits were found to be most pronounced for distal (dexterous) tasks. These tasks—lift can, lift pencil, lift paper clip, stack checkers, flip cards, turn a key in a lock, fold towel, and lift basket—nearly always involve object manipulation and inherently require dexterous motor control of the hands. Sunderland et al. (4) demonstrated that early on after a stroke, spatial accuracy in dexterity tasks performed with the ipsilesional hand correlated with cognitive deficits, such as apraxia, in individuals with LHD. While individuals included in this study did not exhibit severe apraxia and were ~5 years post-stroke, it is possible that mild cognitive deficits, including apraxia, may have impacted dexterous task performance in those with LHD, especially in the more severe ranges of UEFM. Furthermore, we note that our evaluation of dexterous task performance was through timed tests, and not quality of movement or accuracy. It has been suggested that the left hemisphere plays an important role in regulating the timing and speed of movements (13), and thus, injury to the left hemisphere, such as to premotor and fronto-parietal networks (28, 29) may impair planning and sequencing required for smooth and rapid performance of dexterous motor tasks.

### The Role of the Left Hemisphere in the Control of Both Hands

Our main observation that deficits in ipsilesional hand motor capacity scale with contralesional impairment only in LHD is qualitatively similar to previous clinico-behavioral [e.g., (16, 17, 30, 31)] and phenomenological evidence [e.g., (32–34)]. These findings are consistent with a rather simplified organizational model of the nervous system in which certain aspects of motor and/or cognitive control are lateralized to the left (or dominant) hemisphere, such that damage to the left hemisphere results in deficits in skilled motor actions of both upper extremities. For example, using EMG recordings of homologous muscles in the arm, Cernacek (35) demonstrated that the frequency of motor irradiations, i.e., unintended motor output in the ipsilateral hand, were significantly higher from the dominant to the non-dominant extremity. Similarly, Wyke (17) reported that while individuals with left-sided cerebral lesions exhibited bilateral motor deficits in speed and limb postural control, deficits in those with right cerebral lesions were restricted to the contralateral limb. Even within the LHD patient group, the nature and extent of ipsilesional deficits has been shown to be modulated by the degree of (clinical) paresis. For example, Haaland et al. (36) demonstrated that deficits in ipsilesional torque amplitude specification were statistically significant in LHD patients with contralesional upper extremity paresis compared to those with no paresis, despite all other features of the movement and lesion (e.g., error, speed, lesion volume) being similar.

Lastly, in one of the earliest experiments using functional MRI, Kim et al. (37) showed that the task-evoked activation of the left hemisphere was substantially greater for ipsilateral movements compared to the right hemisphere. In later years, a number of neuroimaging (30, 31) and neurophysiologic (32–34) studies have provided confirmatory evidence for the role of the dominant hemisphere in organizing motor outputs to both hands. Our results of co-varying deficits between the contralesional and ipsilesional hand in LHD provides further empirical support for the role of the left hemisphere (in our pre-morbidly right-handed group) in the control of both hands.

### Limitations and Future Considerations

In interpreting our findings, it is important to consider that this study is a retrospective analysis of a relatively small dataset. Therefore, sample size, and characteristics such as the range of impairment, chronicity, age, were limited to what was available. To this point, we conducted analyses with and without outliers, and found that while exclusion of outliers affected the strength of the overall model, it did not affect the probability associated with rejecting the null hypothesis. A prospective study or independent validation in a separate cohort would be ideal, if larger samples were available. A larger sample would render more robust findings that are less sensitive to distortions from outlying values.

Along this line, UEFM scores for the RHD group were restricted toward the more severe range, with the most severely impaired individual's score being 28. This restriction, however, was less so in the LHD group (min. UEFM = 19). Although this limitation in range was circumvented by using group-wise z-scores, we are cautious in generalizing our observations regarding the interaction effect to more severe ranges of motor impairment in RHD. This is quite apparent in the variability around our estimated linear fits especially toward the extreme ranges of predictor values for RHD. Indeed, it is possible that for the more severe range in RHD, there exists a linear relationship between contralesional and ipsilesional motor deficits as illustrated in Figure 1C. Thus, while we can, with some confidence, reject the null hypothesis, our data are insufficient to differentiate between the two alternate hypotheses, and warrant a follow-up study.

Finally, it must be emphasized that the absence of a relationship with contralesional impairment in RHD should not be taken to mean that ipsilesional deficits are absent in this group. In fact, there is substantial evidence to the contrary (38–41). Comparison with an appropriate control group would be necessary to demonstrate the presence of ipsilesional deficits in RHD and the functional implications of these deficits. As alluded to earlier, measuring the speed of performance, as in the case of timed functional tasks assessed here, does not provide specific information about perceptual errors, spatial accuracy or visuomotor deficits, which, based on previous evidence (42, 43), might be a more important component of motor performance in RHD.

## Conclusion

In summary, our results suggest that ipsilesional motor deficits co-vary with the degree of impairment in LHD, but this relationship is less pronounced in RHD. This observation further underscores the extensive motor experiences of the pre-morbidly dominant ipsilesional limb and the importance of the left hemisphere in the control of timed tasks for both hands. For the future, we propose that a hypothetical model of bilateral deficits in LHD is readily testable through a prospective study that uses a bimanual experimental paradigm with sensitive kinematic measures. Such a paradigm could offer important insights into the role and organization of each hemisphere for the control of uni- and bi-manual movements.

## Data Availability Statement

The complete raw dataset along with a codebook for analysis is available through the first author's OSF repository: https://osf.io/pbtk9.

## Ethics Statement

The studies involving human participants were reviewed and approved by Institutional Review Board for the Health Science Campus of the University of Southern California. The participants provided their written informed consent to participate in this study.

## Author Contributions

RV conceptualized the hypothesis, analyzed the data, and drafted the manuscript. CW provided critical review and interpretation of the findings and was involved in manuscript revision.

### Conflict of Interest

The authors declare that the research was conducted in the absence of any commercial or financial relationships that could be construed as a potential conflict of interest.
