# Supplementary material for: Relationship Between Motor Capacity of the Contralesional and Ipsilesional Hand Depends on the Side of Stroke in Chronic Stroke Survivors With Mild-to-Moderate Impairment
Source: Front Neurol. 2020 Jan 8;10:1340. doi: 10.3389/fneur.2019.01340 (PMC6961702; doi:10.3389/fneur.2019.01340)
Supplement: Supplementary file 1 [file Table_1.DOCX]

**Supplementary Materials**

***Inclusionary and Exclusionary Criteria for the DOSE study*** (Winstein et al, 2019, Stroke)

Participants were included if they were: 1) between 21-75 years of age, 2) pre-morbidly right-handed, 3) ≥ 150 days post stroke (chronic phase), 4) had mild to moderate residual motor impairment (Upper Extremity Fugl-Meyer, UEFM score ≥ 19) with mostly resolved upper extremity paresis. Participants were excluded if they had: 1) severe sensory disturbances (no response to light touch or complete loss of proprioception as indicated by the UEFM), 2) current major depressive disorder (score > 3 on PHQ2, depression screening survey) 3) a history of recent surgeries, significant orthopedic injuries, or pain affecting the upper extremity that would restrict shoulder and elbow movement, 4) severe cognitive deficits such as aphasia, apraxia or neglect that would preclude participants from comprehending test instructions or questionnaires.

***Data and Code Availability***

The complete raw dataset along with a codebook for analysis is available through the first author’s OSF repository: <https://osf.io/pbtk9>
